# Differences in Risk Factors and Prevalence of Vascular Calcification between Pre-Dialysis and Hemodialysis Balkan Nephropathy Patients

**DOI:** 10.3390/medicina54010004

**Published:** 2018-03-19

**Authors:** Nenad Petković, Siniša Ristić, Jelena Marinković, Radmil Marić, Marijana Kovačević, Ljubica Djukanović

**Affiliations:** 1 Fresenius Medical Care Dialysis Center, 76230 Šamac, The Republic of Srpska, Bosnia and Herzegovina; nenadpetkovic00@gmail.com; 2 Faculty of Medicine, University of East Sarajevo, 73300 Foča, The Republic of Srpska, Bosnia and Herzegovina; risticsinisa@yahoo.com (S.R.); maricr@yahoo.com (R.M.); kovacevicvmarijana@gmail.com (M.K.); 3 School of Medicine, University of Belgrade, 11000 Belgrade, Serbia; marinkovic.j@gmail.com

**Keywords:** Balkan endemic nephropathy, hemodialysis, pre-dialysis patients, vascular calcification

## Abstract

*Aims*: The aim of this study was to compare the risk factors and prevalence of vascular calcification (VC) in pre-dialysis and hemodialysis (HD) patients with Balkan endemic nephropathy (BEN) or other kidney diseases (non-BEN). *Materials and Methods*: The study involved 115 patients, 32 pre-dialysis and 83 HD patients, separated into groups of BEN and non-BEN patients. In addition to interviews, objective examinations and laboratory analyses, VC was assessed using Adragao score. *Results*: Patients with BEN were significantly older in both groups, while pre-dialysis BEN patients had significantly lower systolic blood pressure, serum cholesterol and phosphorus levels, but higher urinary excretion of phosphorus than non-BEN patients. These differences were lost in HD groups. In pre-dialysis patients, prevalence of VC was lower in BEN than in non-BEN group and mean VC score differed significantly between them (2.8 (1.7) vs. 4.6 (1.8); *p* = 0.009). No significant difference in VC score was found between BEN and non-BEN patients on HD. Multivariate analysis showed that in pre-dialysis patients VC score >4 was associated with lower iPTH and higher serum cholesterol level, but in the HD group with higher serum triglyceride level and longer HD vintage. *Conclusions*: Lower prevalence of risk factors for VC in the BEN than non-BEN patients was found in pre-dialysis but not in HD group and this was reflected in the prevalence and severity of VC in the groups. Prevalence of VC and mean VC score were significantly lower in pre-dialysis BEN than in non-BEN patients but not for those on HD.

## 1. Introduction

Balkan endemic nephropathy (BEN) is a familial, slow progressive tubule-interstitial disease characterized by tubular proteinuria, mild hypertension and in the advanced stage by severe anemia [1]. For BEN patients, data on chronic kidney disease-mineral and bone disorder (CKD-MBD) as well as findings concerning vascular calcification (VC) are scarce and insufficient [2,3,4]. Hyperphosphatemia, one of the main pathogenic factors of VC (as proven in many studies over the past decades [5,6]), has been found more rarely in BEN than in other chronic kidney diseases [2]. Therefore, it was proposed that the prevalence of VC is lower in BEN than in other kidney diseases. Our previous study showed that pre-dialysis BEN patients had a significantly lower VC score than patients with other kidney diseases, but it was not examined in BEN patients on regular hemodialysis (HD).

In the present study the risk factors and prevalence of VC in both HD and pre-dialysis BEN patients was examined with the aim to compare the risk factors and prevalence of VC in pre-dialysis and HD patients with BEN and those with other kidney diseases (non-BEN).

## 2. Materials and Methods

### 2.1. Patients

The study involved 115 patients in the fifth stage of chronic kidney disease. Thus, 32 successive pre-dialysis patients were included during preparation for treatment with regular HD between June and December 2013, while 83 patients on regular HD joined the study in November 2013. The pre-dialysis group consisted of 15 patients with BEN as the primary kidney disease and 17 patients with other kidney diseases, glomerulonephritis (6), diabetic nephropathy (4), pyelonephritis (4) and three with other kidney diseases. All of them started regular HD within four weeks of inclusion in the study. The group of HD patients consisted of all patients receiving HD in the FMC Dialysis Center in Šamac, Bosnia and Herzegovina. They had been on regular HD for 51.8 ± 36.5 months and were dialyzed thrice weekly for 4 h using dialyzers with polysulfone membranes (1.3–1.6 m^2^) and bicarbonate-buffered dialysis solution. Among them were 47 patients with BEN and 36 with other kidney diseases—pyelonephritis (14), glomerulonephritis (5), diabetic nephropathy (5), hypertension (3) and nine with other kidney diseases. We diagnosed BEN using a recently defined consent statement [7] as described elsewhere [8].

All patients were interviewed and an objective examination was carried out in which the following data were registered: age, gender, blood pressure, body mass index (BMI), history of previous cardiovascular disease and smoking habit.

The Ethics Committee of the Foča Medical Faculty, University of East Sarajevo evaluated and approved this study (No. EK-Th 012/13), and all patients gave their informed consent.

### 2.2. Laboratory Analyses

Serum levels of calcium, phosphorus, alkaline phosphatase, iron, total cholesterol, LDL cholesterol and triglycerides as well as hemoglobin were measured by standard laboratory methods. Using a Cobas 6000 (Roche Diagnostics GmbH, Mannheim, Germany) serum level of intact parathyroid hormone (iPTH) was determined by an immuno-chemiluminescent procedure (normal value: 15–65 pg/mL) and 25-hydroxyvitamin D3 (25OHD3) by electrochemiluminescence immunoassay (ECLIA) (normal value: 75–250 ng/mL). Estimated glomerular filtration rate (eGFR) was calculated by the equation proposed by Modification of diet in renal disease study group (MDRD): eGFR = 175 × serum-creatinine-1.154 × age − 0.203 × 0.742 (women) [9]. Residual renal function was calculated using 24-h diuresis as recommended by the European best practice guidelines [10].

### 2.3. Radiological Examinations

VCs in the iliac, femoral, radial and digital arteries were evaluated by one experienced radiologist (S.R.) in plain radiographic films of the pelvis and hands of all examined patients. The simple VC score was calculated as described by Adragao et al. [11]. Briefly, radiographic films of the pelvis were divided into four sections by two lines: a horizontal line over the upper limit of both femoral heads and a vertical line along the middle of the vertebral column. The films of each hand were divided by a horizontal line over the upper limit of the metacarpal bones. The presence of linear calcifications in each part of the film was registered as 1 and their absence as 0. The VC score was the sum of findings from all parts of the films and ranged from 0 to 8.

### 2.4. Statistical Analysis

Continuous variables are presented as mean and standard deviation since their normality assumption has been previously verified using the Kolmogorov-Smirnov test. Categorical variables are presented as frequencies. To assess differences between BEN and non-BEN patients Student’s *t*-test, chi-square, or Fisher’s exact test were used depending on the variables compared. The difference between the compared groups was statistically significant when *p* < 0.05.

We applied univariate and multivariate logistic regression to access factors associated with VC. The following variables: age, gender, diagnosis, HD vintage (only for the HD group), smoking, BMI, systolic and diastolic blood pressure, eGFR (only for the pre-dialysis group), residual renal function (only for the HD group), serum calcium, phosphorus, iPTH, 25OHD3, total cholesterol, LDL-cholesterol, triglycerides, albumin, and CRP were independent variables. The dependent variable was VC score as a binary variable coded as 0 for VC scores ≤4 and as 1 for VC scores >4. Statistical significance was set at *p* < 0.05.

All analyses were performed using the SPSS statistical software package (Version 21; SPSS). IBM Corp. Released 2012. IBM SPSS Statistics for Windows, Version 21.0. Armonk, NY, USA: IBM Corp.

## 3. Results

Table 1 presents the main characteristics of the patients. Patients with BEN were significantly older than those with other kidney diseases in both groups. Moreover, pre-dialysis patients with BEN had lower systolic blood pressure (*p* = 0.014) than non-BEN patients. No significant differences were found between both pre-dialysis and HD groups of BEN and non-BEN patients for BMI, diastolic blood pressure, history of cardiovascular disease and smoking habit.

The results of laboratory analyses for the patients examined are given in Table 2. Estimated GFR was similar in pre-dialysis BEN and non-BEN patients, as was the case with residual renal function in HD patients. Pre-dialysis BEN patients had significantly lower serum levels of phosphorus and cholesterol and higher urinary excretion of phosphorus than those with other kidney diseases. These parameters did not differ between the BEN and non-BEN groups of HD patients and significant disparity was found only in serum iron levels.

**Figure 1:**
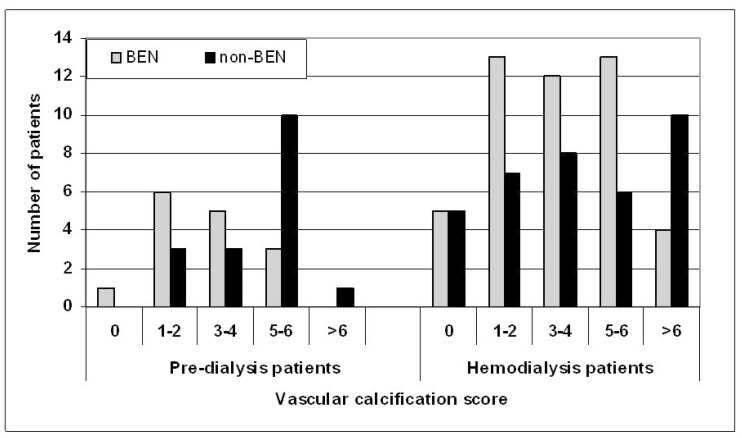
Distribution of pre-dialysis and hemodialysis patients with Balkan nephropathy (BEN) and other kidney diseases (non-BEN) according to vascular calcification score.

Figure 1 presents the distribution of patients according to VC score. In the pre-dialysis group, VC score >4 was detected in 3/15 BEN and 11/17 non-BEN patients (*p* = 0.011). In addition, the mean VC score between BEN and non-BEN pre-dialysis patients differed significantly (2.8 (1.7) vs. 4.6 (1.8); *p* = 0.009). In the HD patient groups, the small differences between BEN and non-BEN patients for distribution according to VC score and mean VC score (3.5 (2.2) vs. 4.1 (2.8), *p* = 0.291) were not statistically significant.

Using multivariate analysis, we showed that in pre-dialysis patients, a VC score of >4 was associated with lower iPTH and higher serum cholesterol levels, but in the HD group, it was associated with a higher serum triglyceride level and longer HD vintage (Table 3). Multivariate analysis that included variables of both pre-dialysis and HD patients indicated that serum cholesterol and LDL-cholesterol levels were associated with a VC score. Patients with a higher cholesterol level and a lower level of LDL-cholesterol were at a higher risk of developing a VC score of >4.

## 4. Discussion

In this study, the prevalence of VCs and factors that may contribute to their occurrence in pre-dialysis and HD patients with BEN or other kidney diseases were compared. Pre-dialysis BEN patients were older, had significantly lower systolic blood pressure, serum levels of phosphorus and cholesterol and higher urinary excretion of phosphorus than non-BEN patients. In the HD group, all these differences between BEN and non-BEN patients, except the difference in age, were lost. These differences in the presence of risk factors for VC between pre-dialysis and HD patients affected the severity of VC in these groups. VC score was significantly lower in BEN than in non-BEN pre-dialysis patients but not in HD patients. Logistic regression analysis showed that, in addition to serum lipids, which were found to be predictors of VC in both pre-dialysis and HD patients, iPTH was a predictor of VC in pre-dialysis and HD vintage in HD patients.

High prevalence of VC has been reported both in HD and pre-dialysis patients [12,13,14,15,16,17]. In these studies, the presence of calcifications was evaluated in different localizations as well as by diverse methods that varied in sensitivity and specificity. We used plain X-ray images for calculation of semiquantitative vascular calcification scores as described by Adragao et al. [11]. There are few data on mineral disorders in BEN. Bukvić et al. [2] recorded that hyperphosphatemia appeared very rarely in BEN patients on HD even without use of phosphate binders. Recently, Premužić et al. [3] observed that BEN patients on HD had lower serum levels of phosphorus and iPTH and, although older, had significantly lower arterial stiffness markers (pulse wave velocity and aortic augmentation index). In addition, they found that BEN was the most significant independent negative predictor of arterial stiffness and proposed that later onset of arterial hypertension in the pre-dialysis stage and lower serum phosphorus level could explain these findings. Both of these studies examined HD patients with BEN and did not examine presence of VC in BEN patients. In our previous study, we presented lower prevalence of VC in pre-dialysis BEN patients in comparison with non-BEN patients [4]. Here, we have examined the prevalence of VC in both HD and pre-dialysis BEN patients and compared it with VC in non-BEN patients. In the non-BEN pre-dialysis group, 11 (64.7%) patients had a VC score >4, which was a somewhat higher percentage than that reported by some other authors, who examined peripheral VC in much larger groups of patients in stage 3 and/or 4 of CKD [12,13,14,15]. In the group of BEN pre-dialysis patients, only three out of 15 (20%) had a VC score >4. This is far less than the relative number of patients with other kidney diseases recorded here and by other authors. In the group of HD patients, a similar number of BEN (17–36%) and non-BEN (16–44%) patients had VC scores >4. These percentages are comparable to the findings of others who used similar methods for the evaluation of VC [11,15,17].

Searching for the cause of the difference in VC prevalence between pre-dialysis and HD patients with BEN, we analyzed risk factors for VC. Different risk factors for VC have been selected in a number of studies and all led to the opinion that risk factors for VC in CKD patients comprised not only traditional ones (older age, male gender, hypertension, diabetes, dyslipidemia, inflammation) but also factors specific for CKD (disorders of mineral metabolism and their regulatory hormones, excessive use of calcium salts as phosphate binders, malnutrition, low levels of VC inhibitors) [18,19,20]. Analysis of the risk factors registered here showed that the only pronounced risk factor in pre-dialysis BEN patients was older age. Several other risk factors (systolic blood pressure, serum level of phosphorus, partly caused by higher urinary excretion of phosphorus, and cholesterol) were lower in BEN than non-BEN patients. These differences in risk factors could explain the disparity in the prevalence of VC between BEN and non-BEN pre-dialysis patients. Multivariate logistic regression analysis confirmed that in pre-dialysis patients, VC score >4 was associated with lower serum iPTH and higher cholesterol levels, but BEN pre-dialysis patients just had higher serum iPTH and lower cholesterol levels as compared with non-BEN patients. On the other hand, all the above-mentioned risk factors, except age, were similar in BEN and non-BEN patients on HD. Our HD patients were on regular HD for four years on average and the severity of VC risk factors arose more from prolonged end-stage renal disease than whether the primary disease was BEN or non-BEN. Multivariate analysis revealed that in the HD group, VC score >4 was associated with longer HD vintage. Residual renal function in both groups of HD patients was minimal, their urinary excretion of phosphorus was similarly low and serum phosphorus level in two HD groups was similar too. In addition, calcium-containing phosphate binders are only available in our country but there is agreement that they are associated with an increase in VC [21,22]. Loss of kidney function also influenced blood pressure, which was similar in both HD groups. This equivalent prevalence of risk factors in BEN and non-BEN patients on HD led to similar prevalence of VC and similar VC scores.

Our study has some limitations. The relatively small number of participants, especially the pre-dialysis group, limited evaluation of VC predictors. In addition, the radiographic method to detect VCs was a simple X-ray imaging technique, so early stages of calcification could have been missed. As in pre-dialysis patients, higher VC was associated with lower iPTH levels, it could be assumed that most patients had a dynamic bone disease rather than osteitis fibrosa. Although this assumption has already been mentioned [3,4], we did not have the opportunity to check it with a bone biopsy. Despite these limitations, for the first time, we have analyzed the prevalence of VC in BEN pre-dialysis and HD patients and detected a significant difference in risk factors and prevalence of VC between BEN and non-BEN pre-dialysis patients as well as between BEN pre-dialysis and HD patients.

## 5. Conclusions

Several risk factors for VC (systolic blood pressure, serum cholesterol and phosphorus levels) were less prominent in pre-dialysis BEN than in non-BEN patients but such differences were not found between BEN and non-BEN patients on HD. This difference in risk factors was reflected in the prevalence and severity of VC in the groups; prevalence of VC and mean VC score were significantly lower in pre-dialysis BEN than in non-BEN patients but this difference between BEN and non-BEN patients was lost in the HD group. Serum iPTH and cholesterol were selected as VC predictors in pre-dialysis patients. Serum triglyceride and HD vintage were selected as VC predictors in HD patients.

As pre-dialysis BEN patients had a lower prevalence of risk factors for VC and consequently VC, exploring strategies for prevention of VC after beginning HD treatment in these patients requires more attention.

## Figures and Tables

**Table 1:** Basic characteristics of the studied patients.

	Pre-Dialysis Patients			Hemodialysis Patients		
	BEN (n = 15)	non-BEN (n = 17)	*p* *	BEN (n = 47)	non-BEN (n = 36)	*p* *
Males, n	11	9	0.234	23	25	0.905
Age, years	71.7 (6.1)	54.7 (11.1)	<0.0001	74.7 (5.7)	64.5 (15.0)	0.0001
Systolic BP, mmHg	133.5 (22.3)	151.5 (14.5)	0.014	150.5 (19.5)	150.5 (22.1)	0.993
Diastolic BP, mmHg	76.9 (8.9)	79.2 (8.1)	0.439	70.7 (10.8)	71.4 (11.8)	0.785
BMI, kg/m^2^	25.0 (3.2)	24.8 (5.1)	0.903	25.2 (4.9)	23.9 (4.7)	0.237
History of						
MI, n (%)	0	3 (17.6)	0.137	2 (4.3)	4 (11.1)	0.232
CVI, n (%)	0	1 (5.9)	0.531	1 (2.1)	1 (2.8)	0.848
PVD, n (%)	2 (13.3)	2 (11.8)	0.675	11 (23.4)	1 (2.8)	0.149
Smoking, n (%)	2 (13.3)	5 (29.4)	0.254	1 (2.1)	3 (8.3)	0.191
HD vintage, months		n.a.		53.7 (31.8)	49.5 (42.6)	0.608

Values are mean (standard deviation) unless otherwise indicated. * *p*—for statistical significance of difference between BEN and non-BEN group according to Student’s *t*-test, chi-square, or Fisher’s exact test as appropriate. BP—blood pressure, BMI—body mass index, MI—myocardial infarction, CVI—stroke, PVD—peripheral vascular disease, n.a.—not applicable.

**Table 2:** Biohemical findings in the examined groups of patients.

	Pre-Dialysis Patients			Hemodialysis Patients		
	BEN	non-BEN	*p* *	BEN	non-BEN	*p* *
Creatinine, μmol/L	658.3 (153.5)	662.2 (99.1)	0.586	608.0 (11.03)	595.5 (176.3)	0.695
eGFR **, mL/min/1.73 m^2^	14.5 (2.8)	7.5 (1.5)	0.759	1.2 (1.6)	1.0 (1.7)	0.658
Calcium, mmol/L	2.40 (0,24)	2.33 (0,15)	0.298	2.51 (0.19)	2.43 (0.23)	0.134
Phosphorus, mmol/L	1.32 (0.36)	1.65 (0.35)	0.015	1.48 (0.38)	1.5 (0.47)	0.843
iPTH, pg/mL	188.6 (105.4)	150.0 (115,6)	0.331	130.5 (140.5)	157.4 (166.7)	0.287
25OHD3, ng/mL	51.1 (22.1)	53.0 (23.1)	0.809	50.9 (33.0)	44.3 (24.4)	0.395
Hemoglobin, g/L	114.3 (10.5)	116.1 (8.1)	0.575	114.1 (11.1)	115.7 (16.1)	0.599
Iron, μmol/L	13.6 (5.3)	15.3 (6.6)	0.446	18.2 (6.2)	14.5 (6.1)	0.011
Albumins, g/L	40.6 (3.3)	41.3 (2.2)	0.521	42.3 (2.3)	41.3 (5.0)	0.252
CRP, mg/L	10.2 (12.6)	11.3 (9.2)	0.782	8.0 (9.0)	14.5 (30.7)	0.174
Cholesterol, mmol/L	4.3 (1.1)	5.2 (0.8)	0.049	5.0 (1.0)	4.8 (1.3)	0.346
LDL-cholesterol, mmol/L	2.4 (0.8)	2.6 (0.6)	0.457	2.7 (0.8)	2.7 (1.0)	0.787
Triglycerides, mmol/L	1.9 (0.9)	2.3 (0.8)	0.134	2.3 (1.4)	2.0 (1.3)	0.346
U-phosphorus, mmol/24 h	6.9 (2.8)	2.9 (0.9)	0.002	1.6 (2.4)	1.2 (2.7)	0.663

Data are presented as mean (SD); * *p*-for statistical significance of difference between BEN and non-BEN group according to Student’s *t*-test. ** eGFR-glomerular filtrate rate: calculated by Modification of Diet in Renal Disease equation in pre-dialysis patients and estimated as the mean of urea and creatinine clearance using urine collections in HD patients, iPTH—intact parathyroid hormone, 25OHD3—25-hydroxyvitamin D3, LDL—low-density lipoprotein; U-phosphorus—urinary phosphorus.

**Table 3:** Variables associated with vascular calcification score (multivariate logistic regression analysis).

Variable	Pre-Dialysis Patients		Hemodialysis Patients		All Patients	
	OR(CI)	*p*	OR(CI)	*p*	OR(CI)	*p*
iPTH, pg/mL	0.99 (0.97–1.00)	0.052	1.00 (0.997–1.004)	0.723	1.00 (0.997–1.003)	0.936
Cholesterol, mmol/L	8.33 (1.53–45.38)	0.014	1.02 (0.24–4.39)	0.980	2.12 (1.30–3.46)	0.003
LDL-cholesterol, mmol/L	0.52 (0.11–2.53)	0.417	1.11 (0.63–1.98)	0.715	0.29 (0.09–0.88)	0.027
Triglycerides, mmol/L	2.63 (0.11–61.49)	0.548	1.86 (1.09–3.18)	0.024	1.27 (0.80–2.00)	0.306
HD vintage, months	n.a.		1.02 (1.00–1.03)	0.029	n.a.	

iPTH—intact parathyroid hormone; LDL—low-density lipoprotein; HD—hemodialysis; n.a.—not applicable.

## References

[1] Djukanovic L., Radovanovic Z., DeBroe M.E., Porter G.A. (2008). Balkan nephropathy. Clinical Nephrotoxins. Renal Injury from Drugs and Chemicals.

[2] Bukvić D., Stefanović D., Milić M., Marić I., Djukanović L. (2003). Hyperphosphatemia appears infrequently in Balkan endemic nephropathy patients on maintenance hemodialysis. BANTAO J..

[3] Premuzic V., Leko N., Stipancic Z., Teskera T., Vinkovic M., Barisic M., Karanovic S., Lela I.V., Dika Z. (2015). Arterial stiffness in patients with endemic nephropathy undergoing hemodialysis. J. Hypertens..

[4] Petković N., Marić R., Gajanin R., Batinić D., Ćuk M., Ristić S., Djukanović L. (2016). Prevalence and risk factors ofvascular calcification in pre-dialysis patients with Balkan endemic nephropathy. Srpski Arhiv za Celokupno Lekarstvo.

[5] Jono S., McKee M.D., Murry C.E., Shioi A., Nishizawa Y., Mori H., Giachelli C.M. (2000). Phosphate regulation of vascular smooth muscle cell calcification. Circ. Res..

[6] Kendrick J., Chonchol M. (2011). The role of phosphorus in the development and progression of vascular calcification. Am. J. Kidney Dis..

[7] Jelaković B., Nikolić J., Radovanović Z., Nortier J., Cosyns J.P., Grollman A.P., Bašić-Jukić N., Belicza M., Bukvić D., Čavaljuga S. (2014). Consensus statement on screening, diagnosis, classification and treatment of endemic (Balkan) nephropathy. Nephrol. Dial. Transplant..

[8] Djukanović L., Djordjević V., Ležaić V., Cukuranović R., Marić I., Bukvić D., Marinković J., Cukuranović J., Rajić M., Stefanović M. (2013). Urinary protein patterns in patients with Balkan endemic nephropathy. Int. Urol. Nephrol..

[9] Levey A.S., Bosch J.P., Lewis J.B., Greene T., Rogers N., Roth D. (1999). A more accurate method to estimate glomerular filtration rate from serum creatinine: A new prediction equation. Modification of diet in renal disease study group. Ann. Intern. Med..

[10] Davison A.M. (2002). European Best Practice Guidelines for Haemodialysis (Part 1). Nephrol. Dial. Transplant..

[11] Adragao T., Pires A., Lucas C., Birne R., Magalhaes L., Gonçalves M., Negrao A.P. (2004). A simple vascular calcification score predicts cardiovascular risk in haemodialysis patients. Nephrol. Dial. Transplant..

[12] Górriz J.L., Molina P., Cerverón M.J., Vila R., Bover J., Nieto J., Barril G., Martínez-Castelao A., Fernández E., Escudero V. (2015). Vascular calcification in patients with nondialysis CKD over 3 years. Clin. J. Am. Soc. Nephrol..

[13] Sigrist M., Bungay P., Taal M.W., McIntyre C.W. (2006). Vascular calcification and cardiovascular function in chronic kidney disease. Nephrol. Dial. Transplant..

[14] Garcıa-Canton C., Bosch E., Ramırez A., Gonzalez Y., Auyanet I., Guerra R., Perez M.A., Fernández E., Toledo A., Lago M. (2011). Vascular calcification and 25-hydroxyvitamin D levels in non-dialysis patients with chronic kidney disease stages 4 and 5. Nephrol. Dial. Transplant..

[15] Lee S.Y., Kim H.Y., Gu S.W., Kim H.J., Yang D.H. (2012). 25-hydroxyvitamin D levels and vascular calcification in predialysis and dialysis patients with chronic kidney disease. Kidney Blood Press. Res..

[16] Damjanovic T., Djuric Z., Markovic N., Dimkovic S., Radojicic Z., Dimkovic N. (2009). Screening of vascular calcifications in patients with end-stage renal diseases. Gen. Physiol. Biophys..

[17] Rosa-Diez G., Bratti G., Filannino G., Peñalba A., Otreras F., Ledesma M., Ortemberg M., Laham G., Vásquez-Durand M., Curcelequi S. (2017). Prevalence of factors related to vascular calcifications in patients with chronic kidney disease ondialysis. Medicina.

[18] Ketteler M., Rothe H., Krüger T., Biggar P.H., Schlieper G. (2011). Mechanisms and treatment of extraosseous calcification in chronic kidney disease. Nat. Rev. Nephrol..

[19] Schlieper G. (2014). Vascular calcification in chronic kidney disease: Not all arteries are created equal. Kidney Int..

[20] Nigwekar S.U., Zhao S., Wenger J., Hymes J.L., Maddux F.W., Thadhani R.I., Chan K.E. (2016). A nationally representative study of calcific uremic arteriolopathy risk factors. J. Am. Soc. Nephrol..

[21] London G.M., Marchais S.J., Guérin A.P., Boutouyrie P., Métivier F., de Vernejoul M.C. (2008). Association of bone activity, calcium load, aortic stiffness, and calcifications in ESRD. J. Am. Soc. Nephrol..

[22] Vervloet M., Cozzolino M. (2017). Vascular calcification in chronic kidney disease: Different bricks in the wall?. Kidney Int..

